# Potential Reporting Bias in Neuroimaging Studies of Sex Differences

**DOI:** 10.1038/s41598-018-23976-1

**Published:** 2018-04-17

**Authors:** Sean P. David, Florian Naudet, Jennifer Laude, Joaquim Radua, Paolo Fusar-Poli, Isabella Chu, Marcia L. Stefanick, John P. A. Ioannidis

**Affiliations:** 1 Department of Medicine, Stanford University School of Medicine, Stanford, USA; 2 Meta-Research Innovation Center at Stanford (METRICS), Stanford University, Stanford, USA; 3 Department of Psychology and Neuroscience, Stanford University School of Medicine, Stanford, USA; 4 Early Psychosis: Interventions & Clinical-detection (EPIC) lab, King’s College London, Institute of Psychiatry Psychology and Neuroscience, London, United Kingdom; 5 FIDMAG Germanes Hospitalaries, CIBERSAM, Saint Boi de Llobregat, Barcelona, Spain; 6 Centre for Psychiatry Research, Department of Clinical Neuroscience, Karolinska Institute, Stockholm, Sweden; 7 OASIS team, South London and the Maudsley NHS Foundation Trust, London, UK; 8 Departments of Health Research and Policy, of Biomedical Data Science, and of Statistics, Stanford University, Stanford, USA

## Abstract

Numerous functional magnetic resonance imaging (fMRI) studies have reported sex differences. To empirically evaluate for evidence of excessive significance bias in this literature, we searched for published fMRI studies of human brain to evaluate sex differences, regardless of the topic investigated, in Medline and Scopus over 10 years. We analyzed the prevalence of conclusions in favor of sex differences and the correlation between study sample sizes and number of significant foci identified. In the absence of bias, larger studies (better powered) should identify a larger number of significant foci. Across 179 papers, median sample size was n = 32 (interquartile range 23-47.5). A median of 5 foci related to sex differences were reported (interquartile range, 2-9.5). Few articles (n = 2) had titles focused on no differences or on similarities (n = 3) between sexes. Overall, 158 papers (88%) reached “positive” conclusions in their abstract and presented some foci related to sex differences. There was no statistically significant relationship between sample size and the number of foci (−0.048% increase for every 10 participants, p = 0.63). The extremely high prevalence of “positive” results and the lack of the expected relationship between sample size and the number of discovered foci reflect probable reporting bias and excess significance bias in this literature.

## Introduction

The nature of possible sex differences in behavior and brain structure and function has been a topic of debate in the scientific community for centuries^1^. Although the presence of Y sex chromosomes affects structural differentiation of some brain regions, such as the sexually dimorphic nucleus of the preoptic area, or “SDN”, in rodents^2,3^, neuroanatomical differences have not been consistently related to robust differences in human brain function^4^. In the field of human neuroimaging research, there are some who argue that sex differences in brain structure, chemistry and function are substantial and widespread^5^, while others claim that there is an overlapping continuum of brain structure and function rather than widespread stereotyped “gendered behavior”^6^. It is also speculated that there may be strong bias and major flaws, particularly in the corpus of neuroimaging literature^7^.

Recent systematic reviews and empirical evaluations of the human neuroimaging and animal studies literature suggest that publication and other reporting biases are prevalent and most studies are underpowered^8^, such that small sample sizes particularly for functional magnetic resonance imaging (fMRI) studies of the brain undermine the reliability and precision of results across the field^9–11^. Specifically, we previously reported evidence of too many statistically significant studies evaluating differences in morphometric measures of regions of interest studies for multiple neurological disease states^12^, and inflated numbers of statistically significant foci in small voxel-based morphometric studies (VBM)^13^ and fMRI studies of the brain^14^.

The goals of the present investigation are to (a) characterize the literature of fMRI studies of the brain that evaluated sex differences and (b) empirically evaluate for evidence of excessive significance bias, which may reflect selective reporting of “positive” (statistically significant) results in this complex and controversial field of neuroscience. The theoretical framework for the present investigation is based on the notion that studies with large samples have more power to detect abnormalities, therefore the number of reported foci should show a positive relationship with the sample size. Small studies should detect only a small proportion of the true signals, whilst larger studies should detect a larger proportion of the true signals. As shown in previous empirical evaluations of neuroimaging studies, a weak or null relationship could indicate potential reporting biases affecting the smaller studies more than the larger studies^11,14^. Moreover, we assessed whether there were any published studies in this field that concluded that there were no statistically significant sex-differences. Given that many studies in the field are very small, a substantial number of studies should find no sex-differences, even if genuine such differences exist. A very low proportion of such “negative” studies would also be cause for concern for similar selective reporting bias.

## Methods

### Inclusion criteria

Articles were included in our analysis if they reported the results of functional magnetic resonance imaging (MRI) studies of human brain to evaluate gender/sex differences. Individual studies were eligible regardless of the topic investigated (task, neurological or psychiatric condition or disease, or other). Exclusion criteria were the following: (i) non-human studies, (ii) studies reporting no direct sex comparison with respect to imaging findings, and (iii) studies that did not report a number of foci. Only English-language publications were included.

### Search strategy

We conducted a four-step literature search. First, we searched on PubMed using the Boolean terms limited to Title and Abstract (“neuroimaging, functional” or “functional brain imaging” or “brain imaging, functional” or “imaging, functional brain” or “fmri” or “mri, functional” or “functional mri”) and (“sex differences” or “sex difference”). Second, we searched on Scopus using the Boolean terms limited to Title and Abstract (“neuroimaging, functional” OR “functional brain imaging” OR “brain imaging, functional” OR “imaging, functional brain” OR “fmri” OR “mri, functional” OR “functional mri” OR “functional magnetic resonance imaging”). All publications listed in PubMed and Scopus over 10 years (between January 1, 2004 and December 31, 2013) were considered. A team of research assistants (EE, EP, EW, IC, KL, RV, SA, SJ) reviewed the abstracts and text of potentially eligible publications for exclusion criteria in double independently. Duplicate publications were eliminated using PMID or DOI. Full texts were retrieved for further scrutiny for all potentially eligible publications. Then the retrieved publications underwent an initial culling of ineligible studies. These publications were then hand searched for inclusion criteria and selected by two analysts independently, with any discrepancies adjudicated until 100% rater agreement was achieved. To achieve a high standard of reporting we have adopted “Preferred Reporting Items for Systematic Reviews and Meta-Analyses” (PRISMA) guidelines^15^.

### Data extraction

The research assistants extracted the total sample size, the year of publication, the type of task (cognitive, motor/somatosensory, resting state fMRI (e.g., task-independent connectivity analyses), the imaging parameters (magnet intensity, slice thickness, degree of smoothing and software packages used), the use of correction (FWE corrected, FDR corrected, unclear correction or no correction), and the possible use of regions of interest (ROI). Data extraction was also performed in double independently by two extractors with any discrepancies adjudicated until 100% rater agreement was achieved.

For the main outcome, two reviewers (JL & FN) identified in each paper the analysis of sex differences that reported the maximum number of foci and extracted this number. Any disagreement was resolved in consultation with a third reviewer (SPD). We also extracted information on whether the authors concluded in the title of the paper or in the abstract that there are no sex differences, i.e. interpreting their results as “negative”.

### Statistical analysis

We followed here the same approach that we used in two previous analyses assessing the relationship between sample size and number of claimed discovered foci^13,14^. Given that studies with large samples have more power to detect differences, the number of reported foci should show a positive relationship with the sample size. A weak or null relationship could thus indirectly indicate potential reporting biases affecting the smaller studies more than the larger studies.

Specifically, the relationship between the number of reported foci in each study and the sample size of the study was assessed with a negative-binomial regression^16^. We used this model instead of a Poisson regression because the distribution of the number of foci showed over-dispersion (mean = 7.2, standard deviation = 7.7). For the sake of completeness, we also conducted simple linear Pearson and non-linear Spearman correlations.

In order to explore experimental variables influencing the relationship between sample size and number of reported foci, sensitivity analyses were conducted on the following subsets of studies: studies published up to and after 2009, studies with up to or more than 32 individuals, studies with up to 80 individuals, studies employing cognitive tasks, studies employing mixed tasks, studies employing motor or somatosensory tasks, resting state fMRI studies, studies conducted in MRI devices with magnets up to or stronger than 1.5 Tesla (T), studies with MRI acquisition slices thickness up to or thicker than 3 mm, studies employing Statistical Parametric Mapping (SPM) or other software packages to pre-process and compare the images, studies applying a smoothing inferior than or of at least 8 mm of full-width at half maximum (FWHM), studies using regions of interest vs. whole-brain analyses, and studies using correction (FWE corrected, FDR corrected, unclear correction or no correction). The sample size of 32 patients was chosen because it has been advocated that the minimum sample size for a neuroimaging study should be 16 patients per group^17^. P-values from subgroup analyses were corrected according to a Bonferroni correction for the number of subgroups assessed (n = 23). All calculations were performed in R.

### Data availability statement

The dataset is available as supplementary information.

## Results

### Database

**Figure 1:**
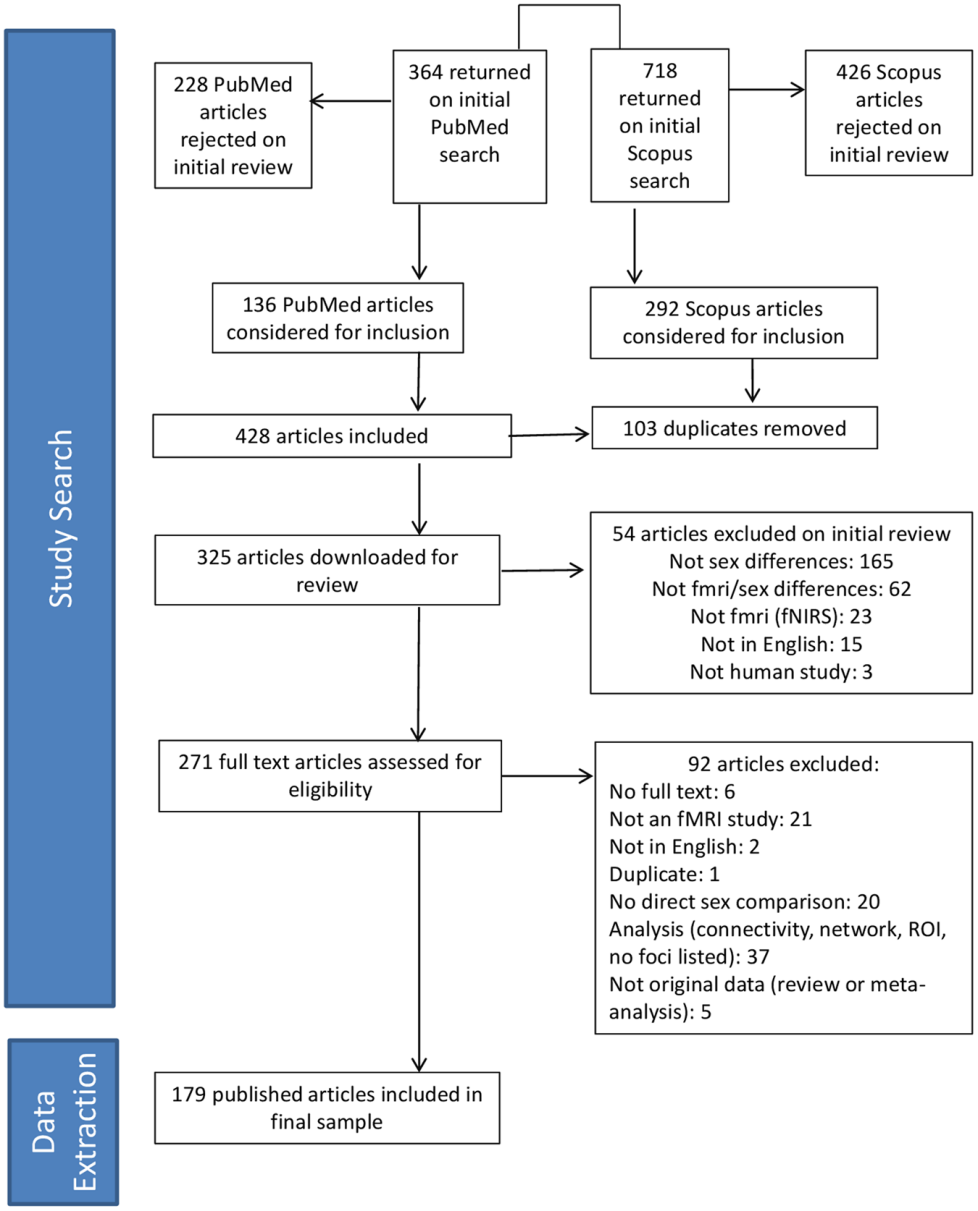
PRISMA Flow chart (Liberati *et al*.^15^) of literature search.

Our literature search identified 1082 references, which were assessed for inclusion criteria. After a first selection based on abstract and title and a check for duplicate or overlapping studies, a final set of 325 individual neuroimaging studies were selected for review of full text articles, resulting in 179 unique studies - constituting the study population. The literature search and the characteristics of the included studies are detailed in Fig. 1 (PRISMA diagram). As shown in Table 1, the number of participants ranged from 8 to 470 across studies (median = 32, 1^st^ quartile = 23, 3^rd^ quartile = 47.5). The number of reported foci per study ranged from 0 to 45 (median = 5, 1^st^ quartile = 2, 3^rd^ quartile = 9.5). 134 studies (75%) reported 10 foci or less in the analysis reporting the greatest number of foci. Other descriptive details of all included studies and by strata of publication year, study size, type of task, and types of imaging and analytic parameters, are depicted in Table 1.

**Table 1:** Number of participants and reported foci in fMRI studies included in the present study.

	Number of studies	Number of participants					Number of foci				
		Min	Q1	Median	Q3	Max	Min	Q1	Median	Q3	Max
All studies	179	8	23	32	47.5	470	0	2	5	9.5	45
**Publication date**											
Studies published up to 2009	90	8	22	26	38	323	0	2	4	8	36
Studies published after 2009	89	10	26	40	64	470	0	2	5	11	45
**Number of patients**											
Studies with up to 32 patients	94	8	19	23.5	26	32	0	2.25	5	10	45
Studies with more than 32 patients	85	33	40	49	74	470	0	2	4	9	36
Studies with up to 80 patients	160	8	22	28.5	40.5	80	0	2	5	9.25	45
**Type of task**											
Studies using cognitive tasks	108	8	24	30.5	44.5	323	0	2	4	9.25	45
Studies using mixed tasks	22	12	24.5	34	47.75	470	0	2.25	5	10.5	36
Studies using motor or somatosensory tasks	38	11	20	30	43	100	0	2	5	9.75	36
Resting state studies	11	16	31	58	122	282	1	3.5	5	7	17
**Magnet intensity**											
Studies with magnets up to 1.5 T	85	10	24	31	42	100	0	2	4	8	31
Studies with magnets stronger than 1.5 T	90	8	22.25	32	56.5	470	0	2.25	5	11	45
**Slice thickness**											
Studies with slices up to 3 mm	53	11	24	32	46	470	0	2	5	11	31
Studies with slices thicker than 3 mm	111	8	23.5	32	49	323	0	2	5	8	45
**Software packages**											
Studies employing SPM	124	11	24	30	47.25	470	0	2	4	8.25	45
Studies not employing SPM	26	11	23.25	35	44.75	158	0	3	5.5	11	31
Degree of smoothing Studies with less than 8 mm smoothing	68	11	22	34	49	470	0	2	5	9.25	36
Studies with 8 mm smoothing or more	97	10	24	30	47	178	0	2	4	8	45
**Use of regions of interest (ROI) or whole brain imaging studies**											
ROI studies	17	14	24	28	48	470	0	1	3	5	26
Whole-brain studies	162	8	22	32	47	323	0	2	5	10	45
**Correction**											
FWE correction	38	10	24	38	65	470	0	2	3.5	8	23
FDR correction	26	18	24	32	47	114	0	2	3.5	11.5	31
No correction	47	8	20	25	33	282	0	4.5	7	11	30
Unclear correction	67	11	24.5	36	49.5	323	0	2	4	7.5	45

Min: minimum, Q1: 1^st^ quartile, Q3: 3^rd^ quartile, Max: maximum.

Some subgroups do not add up to 179 because information required for subgrouping was missing in some studies.

### Studies with “negative” results and conclusions

Of the 179 papers, only two had a “negative” title (“No gender differences in brain activation during the N-back task: an fMRI study in healthy individuals” and “Culture but not gender modulates amygdala activation during explicit emotion recognition”) and found 0 foci. Another three suggest similarities between sexes in their titles (“Females and males are highly similar in language performance and cortical activation patterns during verb generation” and “Comparable cortical activation with inferior performance in women during a novel cognitive inhibition task” and “Sex influences on material-sensitive functional lateralization in working and episodic memory: men and women are not all that different”).

17 (9.5%) papers did not highlight sex differences in their abstracts. Among these, 11 found 0 foci and 6 found some sex differences in analyses that were not highlighted in the abstract. An additional 4 papers found 0 foci, but claimed in the abstract that sex differences were present (based on effects observed in males or in females but without differences observed when genders are compared). The remaining 158 papers (88%) conversely reached “positive” conclusions in their abstract in congruence with reporting some foci related to sex differences in the paper.

### Association between sample size and number of foci in individual fMRI studies

**Figure 2:**
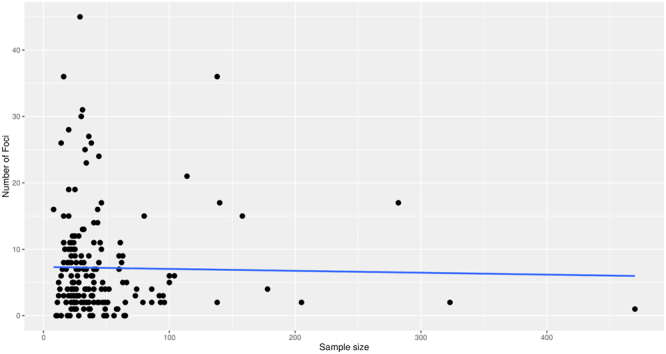
Relationship between sample size and identified number of foci per study.

The median number of foci in small studies (≤32 subjects) (median = 5, range: 0–45) was approximately the same for larger studies (>32 subjects) (median = 4, range: 0–36). There was no statistically significant relationship between sample size and the number of foci in individual studies for all 179 studies using negative binomial regression, Pearson or Spearman correlations (Table 2 and Fig. 2). Nine of the ten studies that had reported the largest number of foci (> = 25) had a sample size <50.

**Table 2:** Relationship between sample size and number of reported foci in subgroups defined by different moderator factors.

	Negative binomial regression		Pearson correlation		Spearman correlation	
	Estimate (a)	P (b)	R	P (b)	Rho	P (b)
All studies	−0.048%	0.630	−0.019	0.601	−0.088	0.878
**Publication date**						
Studies published up to 2009	−0.498%	0.943	−0.115	0.860	−0.083	0.781
Studies published after 2009	−0.030%	0.570	−0.015	0.555	−0.160	0.933
**Number of patients**						
Studies with up to 32 patients	0.705%	0.339	0.041	0.347	0.015	0.442
Studies with more than 32 patients	0.009%	0.479	0.004	0.484	−0.088	0.788
Studies with up to 80 patients	−0.839%	0.947	−0.107	0.912	−0.107	0.911
**Type of task**						
Studies using cognitive tasks	−0.456%	0.964	−0.130	0.911	−0.138	0.923
Studies using mixed tasks	0.004%	0.494	0.001	0.498	−0.054	0.594
Studies using motor or somatosensory tasks	−0.667%	0.780	−0.114	0.751	−0.066	0.654
Resting state studies	0.560%	0.006	0.670	0.012	0.391	0.117
**Magnet intensity**						
Studies with magnets up to 1.5 T	−0.648%	0.873	−0.089	0.792	0.003	0.488
Studies with magnets stronger than 1.5 T	−0.061%	0.658	−0.031	0.615	−0.143	0.910
**Slice thickness**						
Studies with slices up to 3 mm	−0.080%	0.634	−0.028	0.578	−0.015	0.542
Studies with slices thicker than 3 mm	−0.012%	0.525	−0.006	0.523	−0.085	0.814
**Software packages**						
Studies employing SPM	0.102%	0.275	0.040	0.329	−0.061	0.749
Studies not employing SPM	0.261%	0.346	0.106	0.303	−0.015	0.529
**Degree of smoothing**						
Studies with less than 8 mm smoothing	−0.350%	0.965	−0.133	0.860	−0.222	0.966
Studies with 8 mm smoothing or more	0.260%	0.227	0.080	0.218	0.042	0.340
**Use of regions of interest (ROI) or whole brain imaging studies**						
ROI studies	−0.498%	0.903	−0.205	0.785	−0.638	0.997
Whole-brain studies	0.090%	0.304	0.039	0.309	−0.038	0.684
**Correction**						
FWE correction	−0,460%	0.959	−0.197	0.882	−0.199	0.884
FDR correction	0.737%	0.238	0.170	0.204	0.049	0.407
No correction	0.128%	0.330	0.091	0.271	−0.025	0.566
Unclear correction	0.171%	0.246	0.059	0.319	0.021	0.432

P-values reported in the table are uncorrected for multiple comparisons.

^(a)^Increase in the number of reported foci per each increase of 10 patients.

^(b)^P-values were obtained from one-tailed tests

Subgroup analyses did not show any robust relationships between sample size and number of foci when adjusting for multiple corrections (Bonferroni α = 0.05/23 = 0.002). Although not robust to multiple corrections, resting state studies (11 studies) approached significance for negative binomial regression (0.56% foci increase per 10 subjects, p = 0.006) and Pearson correlation (r = 0.67, p = 0.012). For this subgroup, the various association measures were always pointing to a positive correlation.

Furthermore, although subgroup analyses by publication year did not demonstrate the expected relationship, to further explore whether the postulated bias might have improved over more recent years, we conducted a negative binomial regression including sample size and the interaction between sample size and year of publication. This is a post hoc analysis stimulated by external reviewer comments. The interaction had a weak but statistically significant effect (p = 0.009). Specifically, the estimate of the negative binomial regression was found to increase 0.12% each year, with the fitted estimates being −0.968% in 2004 and 0.116% in 2013.

Analyses performed for all other subgroups, were not able to identify any relationship between sample size and number of foci in individual fMRI studies. Of the 132 studies using a correction, cluster-level corrections were made in 30 and voxel-level corrections were made in 32, while this aspect was not specified in 70 studies. Results were similar when we examined the subsets of cluster-level correction studies and of voxel-level correction studies (data not shown in Table 1 because of the unreliability of this variable in our analyses). However, posteriori analyses by cluster-level vs voxel-level did not demonstrate any statistically significant correlation between sample sizes and number of reported foci.

## Discussion

This study explored the potential confounding role of reporting bias in fMRI studies of sex differences by assessing the prevalence of “positive” results and conclusions and whether or not the number of reported foci was positively related to the sample size of the studies. Across 179 identified fMRI studies of the brain published over a decade, few had a title that focused on the lack of sex differences or similarities between sexes and only 17 did not highlight sex differences in their abstract. Given the typically very small sample size of the studies in this literature, this “success rate” is implausibly high. Moreover, there was no statistical correlation between sample size and the number of identified foci. We analyzed relationships across different types of spatial smoothing, slice thickness, date of publication, use of corrected or uncorrected p-values, use of SPM or other statistical approaches, whole brain or ROI studies, and a range of different behavioral and somatosensory tasks. Nonetheless, there was no clear and consistent relationship between sample size and the number of significant foci. These results are surprising because owing to higher statistical power, studies with larger sample sizes should have been able to detect more differences when true sex differences are present^9–11^.

The lack of relationship observed in these analyses may reflect systematic reporting bias in small fMRI studies that produces a published literature with more sex difference signals than truly exist. We have previously reported a small but significantly positive correlation between sample size and number of brain abnormalities in VBM studies with variance by publication date, statistical thresholds and other imaging parameters^13^, and a lack of a consistently positive relationship between sample size and foci across the larger field of published fMRI studies^14^. The median number of foci in small studies (≤32 subjects) was approximately the same for larger studies (>32 subjects). As has been shown for morphometric^12^ and fMRI studies at large^9–11,14^, it appears that there is reporting bias driving an excess of significance. Studies with smaller sample sizes and reduced statistical power have been shown to produce imprecise and frequently spurious false positive results and it is possible that studies and analyses with more significant results are selected for publication. While, this problem is not specific to the study of sex differences but inherent to small-sample fMRI research, this problem might be exacerbated by the very simple fact that subgroup analyses based on sex are always tempting to do and easy to perform (in most datasets, information on sex are generally present). It is probable that the high proportion of “positive findings” result from a combination of factors including publication bias due to journal editorial practices favoring positive results, and significance biases including selective outcome and analysis reporting bias (reporting additional analyses that were not pre-specified), under-reporting of null results (“file-drawer problem”, particularly in underpowered studies), p-value “hacking” (manipulation of the analysis parameters until significant results are obtained), and other factors identified across the psychological literature^18,19^ and in the fMRI literature^8^. We have published suggestions for reducing these practices^8^ and there is some evidence that efforts to promote open science are bearing fruit as more light is shed on these problems^20^. We do not know if these recommendations are now widely followed by researchers, but if investigators are following the recommendation to use more stringent primary thresholds only for higher power studies, this might explain why higher-powered studies are not reporting more foci; if this is rampant and systematic across the field, it would represent a type of reporting or significance bias.

Our results could also reflect a dearth of biologically plausible sexual dimorphism in brain function across a range of many tasks published in the literature. A previous systematic review of fMRI studies concluded that there was widespread publishing of underpowered studies with “false-positive claims of sex differences in the brain, to enable the proliferation of untested, stereotype-consistent functional interpretations”^21^ and suggested that widespread scientific assumptions that female and male brains are functionally distinct, dichotomous, fixed, and invariant due to a sexually differentiated genetic blueprint are not scientifically justified and may be sexist^22^. Other investigators have posited that sex differences in cognitive test performance are explained by hormonal differences throughout development in combination with cultural influences, gender stereotypes, and biopsychosocial interactions^23^; and that females and males belong to a single heterogeneous population rather than two distinct populations with regard to brain structure and function^24^.

Some limitations should be acknowledged. First, in order to prevent any difficulty due to multiple measurements, we extracted foci for the analysis with the largest number of foci. But in many studies there was more than one analysis. As a result, some studies may have claimed to have identified far more significant foci than the number we have extracted. Thus, our analysis probably underestimates the potential problem of having too many statistically significant claims for sex differences in fMRI studies. Second, there were differences in the types of study designs across studies. We attempted to address this methodological heterogeneity with sensitivity analyses across different subgroups defined by methodological features. However, these subgroup analyses might be underpowered to demonstrate the relationship explored. Conversely, the one positive subgroup result encountered may be a spurious association found by chance since it did not survive correction for multiplicity. Third, the statistical significance of the results of fMRI studies may depend on the analytical method used and some parametric methods have been shown to yield inappropriate type I errors^25^. Here, we considered the correction used but did not re-analyze the raw data or to confirm the results using the same assumptions and statistical methods employed by the original authors. In addition, we sought to control for the level of correction (cluster-level vs. voxel-level) in each study. We attempted to extract this information but use of clusterwise vs voxelwise statistical correction was often not clearly documented in the different papers. Another open question that we were not able to control is how to appraise the statistical stringency. That is, is for instance 0.005 cluster-level FWE more or less stringent than 0.01 voxel-level FWE or 0.01 cluster-level FDR and so forth.

Fourth, our literature search was limited to studies published in the decade 2004–2013. Curating the database required extensive time and effort and it was not felt that enough additional information would be gained to justify updating the searchto capture more recent studies at this time. It is unlikely that earlier or more recent studies would present a different pattern, but empirical evaluations of very recent fMRI studies may be worth performing in the future, especially if large, multicenter investigations start appearing more frequently in this literature. Interestingly, we observed a small but statistically significant interaction between sample size and publication year, suggesting that the most recent studies may have operated in an environment where the strength of biases may have decreased.

Fifth, our searches were extensive, but we might have missed some studies of sex differences. In particular, we may have missed some studies that found no significant sex differences and this “negative” result was alluded to only in some fine print in the paper and thus could not be retrieved with our literature searches. If so, this would also be a form of reporting bias, if “positive” results are not only more likely to be published, but are also more prominently presented when published, as compared with “negative” results.

Sixth, we acknowledge that an increase of the sample size and power may enable non-significant voxels between two close clusters to achieve statistical significance, thus sometimes converting the two close clusters into a single larger one. The number of foci should not be affected by this conversion, but some authors choose to report only three foci per cluster. We did not assess reporting of <= 3 foci/cluster in our sensitivity analyses. In such a case, the relationship between the sample size and the number of foci could be downwards biased. However, in a previous publication, we found no evidence of an effect of this practice on the correlation between sample size and number of foci reported^13^. Although this modeling was from a database of VBM studies, it should be noted that in our earlier mega-analysis of fMRI studies^14^, we found the expected relationship between sample size and number of foci in meta-analyses – which also have the same effect of converting close clusters into a single, robust activation focus using activation-likelihood estimation. We may also not have extracted some other important confounders such as study quality defined in other ways. We cannot exclude that some large studies may be of poor quality and thus are less prone to find foci than smaller studies. Nevertheless, it seems unlikely since one would expect higher quality criteria in larger investigations that are typically performed by more experienced teams.

Importantly, our evaluation cannot conclude that there are no biologically plausible functional sex differences in human brain function, cognition or behavior that would be reflected in fMRI studies of the brain. However, the present data suggest that there is likely excess significance bias in the reported results of fMRI studies of sex differences of the brain.

This excess significance and reporting bias may stem from a constellation of factors that are likely to affect more prominently the literature of small studies. These factors include, but are not limited to, lack of pre-registration^8^, large flexibility in the modes of analyses^26^, inappropriate statistical methods^26^ and selection pressure from the current reward and incentives system to report the most significant results^8^. Conversely, solutions to this problem may involve pre-registered protocols and registration databases^8^, openness and transparency with wider data sharing practices such as Neurovault^27^ and OpenfMRI^28^, as well as pre-registered reports^29^ and other efforts that try to minimize selective reporting^20,30^.

## Electronic supplementary material

Supplementary Information
